# Changing treatment patterns for coronary artery revascularization in Canada: the projected impact of drug eluting stents

**DOI:** 10.1186/1471-2261-4-23

**Published:** 2004-12-13

**Authors:** Michael T Halpern, Michael Lacey, Mary Ann Clark, Miguel A Valentin

**Affiliations:** 1 Exponent, Alexandria VA, USA; 2 Boston Scientific Corporation, Natick MA, USA

## Abstract

**Background:**

To evaluate current treatment patterns for coronary artery revascularization in Canada and explore the potential impact of drug eluting stents (DES) on these treatment patterns.

**Methods:**

Eleven cardiologists at multiple Canadian academic centers completed a questionnaire on coronary artery revascularization rates and treatment patterns.

**Results:**

Participating physicians indicated slightly higher rates of PTCA, CABG, and stent implantation than reported in CCN publications. Participants estimated that 24% of all patients currently receiving bare metal stents (BMS) would receive DES in the first year following DES approval in Canada, although there was a large range of estimates around this value (5% to 65%). By the fifth year following DES approval, it was estimated that 85% of BMS patients would instead receive DES. Among diabetic patients, estimates ranged from 43% in the first year following approval to 91% in the fifth year. For all patients currently receiving CABG, mean use of DES instead was estimated at 12% in the first year to 42% at five years; rates among diabetic patients currently undergoing CABG were 17% in the first year to 49% in the fifth year.

**Conclusions:**

These results suggest a continued increase in revascularization procedures in Canada. Based on the panel's responses, it is likely that a trend away from CABG towards PTCA will continue in Canada, and will be augmented by the availability of DES as a treatment option. The availability of DES as a treatment option in Canada may change the threshold at which revascularization procedures are considered.

## Background

Coronary artery disease (CAD) is a common condition in western society [1]. Treatment of CAD often involves surgical revascularization, that is, removal of coronary artery stenoses to restore sufficient myocardial blood flow. Currently, there are three major treatment options for coronary artery revascularization: coronary artery bypass grafting (CABG), percutaneous transluminal coronary angioplasty (PTCA), and coronary artery stenting [2]. Treatment choice is based on a variety of factors, including patient age, comorbidities, extent of disease (i.e., number and location of affected coronary arteries), and disease severity.

Treatment choices and treatment patterns for CAD have changed over the past several years, and are likely to evolve further in the next few years. Drug eluting stents (DES) are a newly available treatment modality. DES are stents that incorporate bioactive coatings (polymer or non-polymer) permitting the release of associated molecules to attenuate the processes of restenosis. Preliminary clinical data suggests that use of DES can substantially reduce the rates of restenosis seen following implantation of bare metal stents but at much lower incidences of severe procedure-related complications as compared to CABG surgery [3,4]. DES may also be an important treatment option for populations such as individuals with diabetes and multi-vessel disease, who appear to have better outcomes with CABG [2].

It is likely that the availability of DES will change treatment patterns for patients with CAD [5]. To better understand current treatment patterns for CAD and the potential role of DES in Canada, we developed and administered a questionnaire to a panel of Canadian cardiologists. This manuscript describes the estimates regarding current and future CAD treatment patterns in Canada provided by this panel.

## Methods

The objective of this study was to obtain opinions and estimates from Canadian cardiologists regarding the annual rates of PTCA, CABG, and stenting in Canada, treatment patterns associated with these procedures, and the potential future impact of DES on coronary revascularization treatment patterns. The results from this questionnaire were used in the development of health economic models used to evaluate these stents for use among Canadian patients.

The questionnaire asked participating physicians to assess the projected annual procedure rates for CABG, PTCA and stent implantations published by the Cardiac Care Network of Ontario (CCN) Target-Setting Working Group [6]. In addition to the estimated rates of the revascularization procedures, respondents were also requested to provide a lower and upper bound for each rate. Additional sections of the questionnaire provided recommendation rates for repeat revascularization procedures (following restenosis) depending on the type of first procedure. For example, physicians were asked for the percentage of patients they would recommend CABG after restenosis following PTCA. In the final sections, the questionnaire requested information on the projected use of DES among patients currently receiving bare metal stents or CABG. The questionnaire instructed respondents that if they agreed with provided reference values (based on published estimates) for a particular item, they could leave that item blank. However, respondents free to provide estimates that were greater, lesser, or unchanged compared to the reference values.

Cardiologists at multiple academic centers distributed across Canada were identified for this study. These physicians were contacted, provided a brief introduction to the study, and offered a study honorarium of $400 dollars to participate. The questionnaire was distributed to physicians willing to participate. Completed questionnaires were collected and data was entered into Excel. Summary statistics including mean, median, maximum, and minimum values were computed for the various estimates provided by the participating physicians. The summary statistics were returned to those doctors who completed questionnaires. Study participants were then asked to comment on the summary results and indicate any questions or comments on the results. At the time of the study, only one drug-eluting stent (CYPHER™, Cordis Corporation) had been approved for use by Health Canada (Nov. 2002).

## Results

### Study participants

A total of 18 Canadian physicians were contacted and invited to participate. Eleven physicians agreed to participate and complete the questionnaire, a response rate of 61%. All eleven physicians were male, specialized in cardiology, and had experience in this specialty ranging from 6 to 30 years. The average length of time specializing in cardiology was 12.8 years. The average age of participating physicians was 44.0 years. In each of the result tables, the number of panel members responding to each item is reported.

### Procedure annual rates

The CCN Target-Setting Working Group (TSWG) projected annual rates of CABG surgery and PTCA of 110 and 160 per 100,000 Canadians, respectively [6]. Based on responses from the questionnaire, participating physicians reported slightly higher mean estimates of 112.7 (range of 100 to 150) for CABG and 172.3 (range of 125 to 200) for PTCA (Table 1). The TSWG also estimated that the rate of stent implantations among PTCA cases is over 90%. Questionnaire results indicated a similar proportion of patients receiving stents (92.1%, range 90% to 99%).

**Table 1:** Estimated rates of CABG, PTCA, and Stenting

	**Mean**	**Median**	**Min**	**Max**	**# of Panel Members Responding**
**Annual rate of CABG per 100,000 **(CCN estimate: 110)					
estimate	112.7	110	100	150	11
not lower than	91.1	90	60	110	9
not higher than	131.1	120	110	190	9
**Annual rate of PTCA per 100,000 **(CCN estimate: 160)					
estimate	172.3	180	125	200	11
not lower than	147.8	150	110	180	9
not higher than	214.4	200	160	300	9
**Percent of PTCA patients receiving stents **(CCN estimate: >90%)					
estimate	92.1%	90.0%	90.0%	99.0%	11
not lower than	81.8%	84.5%	70.0%	90.0%	8
not higher than	96.8%	96.0%	94.0%	100.0%	8

### Recommendations for subsequent revascularization procedures

Table 2 presents results for recommendations from participating physicians for patients who need a subsequent revascularization procedure after having received either stenting or CABG. For patients requiring subsequent revascularization after stenting, PTCA would be recommended for approximately 40% of cases. Brachytherapy was the second most frequently recommended procedure (23%), followed by similar rates for CABG and a second stenting. For revascularization following CABG surgery, stenting was recommended for almost 80% of patients while PTCA and CABG were recommended for 6.8% and 13.6% of patients, respectively.

**Table 2:** Subsequent revascularization procedures following stenting or CABG

	**Mean**	**Median**	**Min**	**Max**	**# of Panel Members Responding**
**Percent of patients receiving each procedure following initial stenting**					
PTCA	38.5%	40.0%	9.0%	75.0%	11
Stenting	18.4%	15.0%	0.0%	65.0%	11
CABG	19.8%	15.0%	1.0%	50.0%	11
Brachytherapy	23.3%	20.0%	0.0%	90.0%	11
**Percent of patients receiving each procedure following initial CABG**					
PTCA	6.8%	5.0%	0.0%	15.0%	11
Stenting	79.5%	80.0%	55.0%	90.0%	11
CABG	13.6%	10.0%	5.0%	40.0%	11

### Projected use of DES among Canadian CAD patients

Tables 3 through 6 present results from the cardiologist panel regarding the projected use of DES once after they are approved in Canada. For these projections, we separately asked the panel members to provide estimates on the proportion of patients currently receiving bare metal stents who would likely receive DES instead versus the proportion currently undergoing CABG who would likely receive DES instead. We also requested rates DES adoption separately for the entire Canadian CAD population versus the subpopulation of Canadian CAD patients with diabetes, as the diabetic population is at higher risk for adverse clinical outcomes [7] and therefore may have different treatment patterns. In all cases, responses for the proportion of patients likely to receive DES were requested for all patients in the specified population as well as separately for patients with single- vs. multi-vessel CAD. Projections were requested annually for the first five years following approval of DES in Canada.

**Table 3:** Estimated Percentage of BMS Patients Likely to Receive DES by Year Following DES Approval

	**Mean**	**Median**	**Min**	**Max**	**# of Panel Members Responding**
**% of Bare Metal Stent Patients Receiving DES, 1st Year Following Approval**					
all patients	24.0%	22.5%	5.1%	65.0%	10
all patients with single-vessel	18.8%	12.6%	5.0%	50.0%	8
all patients with multi-vessel	32.9%	25.0%	5.0%	80.0%	6
**% of Bare Metal Stent Patients Receiving DES, 2nd Year Following Approval**					
all patients	36.6%	40.0%	10.2%	65.0%	10
all patients with single-vessel	28.4%	30.4%	10.0%	50.0%	9
all patients with multi-vessel	43.9%	40.0%	5.1%	100.0%	8
**% of Bare Metal Stent Patients Receiving DES, 3rd Year Following Approval**					
all patients	57.1%	60.0%	20.5%	80.0%	10
all patients with single-vessel	53.5%	60.0%	20.0%	80.9%	9
all patients with multi-vessel	61.2%	65.0%	10.3%	100.0%	8
**% of Bare Metal Stent Patients Receiving DES, 4th Year Following Approval**					
all patients	76.7%	80.0%	50.8%	91.0%	10
all patients with single-vessel	69.6%	80.0%	30.0%	91.0%	9
all patients with multi-vessel	85.0%	80.0%	80.0%	100.0%	7
**% of Bare Metal Stent Patients Receiving DES, 5th Year Following Approval**					
all patients	85.0%	90.0%	49.0%	100.0%	10
all patients with single-vessel	81.7%	90.0%	49.0%	100.0%	9
all patients with multi-vessel	88.4%	90.0%	60.0%	100.0%	8

**Table 4:** Estimated Percentage of Diabetic BMS Patients Likely to Receive DES by Year Following DES Approval

	**Mean**	Median	**Min**	**Max**	**# of Panel Members Responding**
**% of Diabetic Bare Metal Stent Patients Receiving DES, 1st Year Following Approval**					
all patients	43.2%	40.0%	10.2%	90.0%	11
all patients with single-vessel	39.5%	30.0%	10.2%	80.0%	9
all patients with multi-vessel	61.4%	50.0%	30.0%	100.0%	7
**% of Diabetic Bare Metal Stent Patients Receiving DES, 2nd Year Following Approval**					
all patients	60.6%	60.0%	25.5%	100.0%	11
all patients with single-vessel	57.9%	50.0%	25.5%	100.0%	9
all patients with multi-vessel	75.0%	75.0%	40.0%	100.0%	7
**% of Diabetic Bare Metal Stent Patients Receiving DES, 3rd Year Following Approval**					
all patients	77.9%	80.0%	50.0%	100.0%	11
all patients with single-vessel	75.7%	80.0%	50.0%	100.0%	9
all patients with multi-vessel	81.4%	80.0%	50.0%	100.0%	7
**% of Diabetic Bare Metal Stent Patients Receiving DES, 4th Year Following Approval**					
all patients	86.1%	90.0%	60.0%	100.0%	11
all patients with single-vessel	85.2%	90.0%	60.0%	100.0%	9
all patients with multi-vessel	87.1%	90.0%	60.0%	100.0%	8
**% of Diabetic Bare Metal Stent Patients Receiving DES, 5th Year Following Approval**					
all patients	90.9%	90.0%	70.0%	100.0%	11
all patients with single-vessel	88.0%	90.0%	70.0%	100.0%	10
all patients with multi-vessel	89.0%	90.0%	70.0%	100.0%	10

**Table 5:** Estimated Percentage of CABG Patients Likely to Receive DES by Year Following DES Approval

	**Mean**	**Median**	**Min**	**Max**	**# of Panel Members Responding**
**% of CABG Patients Receiving DES, 1st Year Following Approval**					
all patients	12.3%	5.4%	0.0%	50.0%	11
all patients with single-vessel	7.8%	5.1%	0.0%	20.0%	9
all patients with multi-vessel	15.7%	6.0%	0.0%	80.0%	8
**% of CABG Patients Receiving DES, 2nd Year Following Approval**					
all patients	17.5%	12.6%	5.0%	50.0%	10
all patients with single-vessel	9.7%	10.0%	2.0%	20.0%	8
all patients with multi-vessel	21.4%	15.1%	5.0%	80.0%	8
**% of CABG Patients Receiving DES, 3rd Year Following Approval**					
all patients	31.7%	30.0%	5.0%	90.0%	10
all patients with single-vessel	29.1%	27.7%	2.0%	90.0%	8
all patients with multi-vessel	28.9%	25.0%	5.0%	90.0%	8
**% of CABG Patients Receiving DES, 4th Year Following Approval**					
all patients	37.3%	33.0%	5.0%	90.0%	10
all patients with single-vessel	32.0%	30.2%	5.0%	90.0%	8
all patients with multi-vessel	33.2%	30.0%	5.0%	90.0%	8
**% of CABG Patients Receiving DES, 5th Year Following Approval**					
all patients	42.1%	40.2%	5.0%	90.0%	10
all patients with single-vessel	35.1%	30.2%	5.0%	90.0%	7
all patients with multi-vessel	37.6%	30.0%	5.0%	90.0%	7

**Table 6:** Estimated Percentage of Diabetic CABG Patients Likely to Receive DES by Year Following DES Approval

	**Mean**	**Median**	**Min**	**Max**	**# of Panel Members Responding**
**% of Diabetic CABG Patients Receiving DES, 1st Year Following Approval**					
all patients	16.9%	10.0%	1.1%	65.0%	10
all patients with single-vessel	17.4%	5.1%	1.1%	50.0%	7
all patients with multi-vessel	19.5%	10.0%	1.1%	80.0%	9
**% of Diabetic CABG Patients Receiving DES, 2nd Year Following Approval**					
all patients	26.0%	17.5%	5.1%	80.0%	10
all patients with single-vessel	21.3%	15.2%	5.0%	50.0%	8
all patients with multi-vessel	21.1%	15.0%	5.1%	80.0%	9
**% of Diabetic CABG Patients Receiving DES, 3rd Year Following Approval**					
all patients	33.5%	25.0%	10.0%	90.0%	10
all patients with single-vessel	30.1%	25.2%	5.0%	90.0%	8
all patients with multi-vessel	26.3%	17.5%	5.1%	90.0%	9
**% of Diabetic CABG Patients Receiving DES, 4th Year Following Approval**					
all patients	42.6%	40.0%	10.0%	90.0%	10
all patients with single-vessel	37.0%	32.8%	5.0%	90.0%	8
all patients with multi-vessel	34.0%	30.0%	10.2%	90.0%	9
**% of Diabetic CABG Patients Receiving DES, 5th Year Following Approval**					
all patients	48.6%	50.0%	10.0%	90.0%	10
all patients with single-vessel	41.4%	45.0%	5.0%	90.0%	8
all patients with multi-vessel	42.6%	37.5%	15.0%	90.0%	10

Tables 3 and 4 present estimated percentage for all CAD patients and diabetic patients (respectively) who are currently receiving bare metal stents but are likely to receive DES once approved. As presented in Table 3, the mean estimated percentage for all CAD patients in the first year of approval is 24%. A large range was present around this mean, from a minimum of 5.1% to a maximum of 65%. However, the median (22.5%) was similar to the mean, suggesting that outliers did not substantially skew the mean value. Patients with single-vessel disease were less likely to receive DES (18.8%), while those with multi-vessel disease were more likely (32.9%). The proportion of patients projected to receive DES rather than bare metal stents increased with each subsequent year after DES approval. In each year, a greater proportion of multi-vessel disease patients are projected to receive DES than are single vessel disease patients. During the fifth year following approval, the panel estimated that 85% of all bare metal stents patients are likely to receive DES instead. Ranges around the annual mean values continued to be large, with the minimum estimate being 49% and the maximum estimate of 100%.

Projected use of DES among diabetic patients who currently receive bare metal stents is presented in Table 4. Among patients with diabetes, the estimated percentage likely to receive DES is higher than the corresponding values of the overall population. In the first year following DES approval, 43.2% of patients with diabetes who would have received bare metal stents are projected to receive DES instead. While the median proportion of diabetic patients receiving DES in this first year (40%) is similar to the mean, suggesting that outliers do not skew the projections, a very large range of responses was present (10.2% to 90%). The estimated proportion of patients receiving DES rather than bare metal stents in the first year was 39.5% for single vessel disease patients with diabetes, and 61.4% for multi-vessel disease patients. As with the overall population of CAD patients currently receiving bare metal stents (Table 3), the proportion of patients with diabetes receiving DES instead of bare metal stents increases in each subsequent year, and the percentage is greater for multi-vessel disease patients than for single vessel disease patients. In the fifth year following DES approval, it is estimated that 90.9% of patients with diabetes who would have received bare metal stents will instead receive DES (range 70% to 100%).

Table 5 presents estimates from the cardiologist panel for CABG patients who are likely to receive DES after approval. For each year, the proportion of CABG patients who would instead receive DES is approximately half the proportion of bare metal stent patients who would receive DES instead (Table 3). Of all CABG patients, 12.3% are estimated to likely receive DES during the first year following approval in Canada. The range of estimates for receipt of DES rather than CABG was substantial, from a minimum of 0% to a maximum of 50%. The median estimate, 5.4%, is lower than the mean, suggesting that higher estimates may be skewing the mean. In the first year following approval, 7.8% of single vessel disease patients and 15.7% of multi-vessel disease patients would receive DES rather than CABG. In years three through five after DES approval, the estimated proportions of single vessel disease and multi-vessel disease patients likely to receive DES rather than CABG are both less than the proportion among the overall CABG population. This is due to missing data, in that some panel members provided projections only for the overall population and/or one of the population subgroups. In these cases, the relative projections for single vessel and multi-vessel disease patients cannot be directly compared to the estimates for the overall population.

Similar to DES adoption among bare metal stent patients, the likelihood of DES use among CABG patients increases with each year after approval. At year five, 42% of all CABG patients are likely to receive DES compared to 85% of bare metal stent patients. In all years except year three, the proportion of single vessel disease CABG patients instead receiving DES is less than the proportion for multi-vessel disease CABG patients.

Table 6 provides the estimated rates of DES use for patients with diabetes currently receiving CABG. In the first year following approval, 16.9% of patients with diabetes who would have received CABG are projected to likely to receive DES instead. The range of estimates around this value is large (1.1% to 65.0%). The mean estimate of DES adoption among diabetic CABG patients increased each year, and is larger each year than the corresponding mean estimate for the overall population of patients who would receive CABG. However, the estimated proportion of patients with diabetes receiving DES instead of CABG is less than the estimated proportion for bare metal stent patients.

Rates for single and multi-vessel disease patients with diabetes are similar; however, as noted above, missing data makes comparison of these subpopulations to the overall diabetic population difficult. During the fifth year following approval, an estimated 48.6% of diabetic patients who would have received CABG surgery are instead projected receive DES.

### Recommended use of DES among Canadian CAD patients

Tables 3 through 6 present the proportion of bare metal stent and CABG patients who are likely to receive DES rather than these other revascularization procedures. In a final question to the cardiologist panel, we asked for estimates of the proportion of bare metal stent and CABG patients in the overall CAD population who should receive DES rather than these other procedures. In requesting this additional information, the questionnaire specified that respondents could indicate that the proportion of patients who should receive DES is the same as or different from the proportion that are likely to receive DES (as presented in Tables 3 and 5). The questionnaire also specified that in estimating the proportion of patients who should receive DES instead of bare metal stents of CABG, panel members should assume that funding is available for this intervention. Thus, this question addressed the projected use of DES in a best-case scenario, without economic restrictions.

The estimated percentages of patients who should receive DES are summarized in Table 7. During the first year of approval, the mean estimated percentage of bare metal stent patients who should receive DES is 42.8%; this is close to double the estimate of the proportion of bare metal stent patients who are likely to receive DES during the first year following approval (24.0%, Table 3). The estimated percentage of CABG patients who should receive DES during the first year following approval is 16.8%, an increase of 37% over the proportion of CABG patients likely to receive DES that year (12.3%, Table 5). These estimated percentages of patients who should receive DES increase with each year after DES approval. At year five, the panel indicated that 86.8% of bare metal stent patients and 43.7% of CABG patients should be receiving DES. The median responses are very similar to these values, suggesting that outliers are not distorting the presented means. However, while the range around the mean proportion of bare metal stent patients who should receive DES has decreased (minimum 60.8%, maximum 100%), the range around the proportion of CABG patients who should receive DES remains very large (5% to 90%). Thus, there are considerable differences in opinion regarding the appropriate patients to convert from CABG to DES.

**Table 7:** Estimated Percentage of BMS and CABG Patients who Should Receive DES by Year Following DES Approval*

	**Mean**	**Median**	**Min**	**Max**
**% of Patients Who Should Receive DES, 1st Year Following Approval**				
Bare metal stent patients	42.8%	30.0%	5.1%	100.0%
CABG patients	16.8%	10.0%	5.0%	50.0%
**% of Patients Who Should Receive DES, 2nd Year Following Approval**				
Bare metal stent patients	56.0%	50.0%	10.2%	100.0%
CABG patients	25.5%	20.0%	5.0%	80.0%
**% of Patients Who Should Receive DES, 3rd Year Following Approval**				
Bare metal stent patients	70.1%	90.0%	30.0%	100.0%
CABG patients	31.4%	30.0%	5.0%	90.0%
**% of Patients Who Should Receive DES, 4th Year Following Approval**				
Bare metal stent patients	79.7%	90.0%	40.6%	100.0%
CABG patients	37.8%	35.0%	5.0%	90.0%
**% of Patients Who Should Receive DES, 5th Year Following Approval**				
Bare metal stent patients	86.8%	90.0%	60.8%	100.0%
CABG patients	43.7%	40.0%	5.0%	90.0%

*In answering this question, respondents were asked to assume that funding for DES was available. All questions were responded to by all 11 members of the study panel.

## Discussion

This study presents results from a panel of Canadian cardiologists on treatment patterns for coronary artery revascularization and the potential future adoption of DES in these treatment patterns. Previous reports have debated whether the rate of coronary revascularization in Canada is likely to decrease [8] or increase [9] during the present decade. The estimated procedure rates provided by the panel were slightly higher than those from the CCN, suggesting a continued increase in revascularization procedures. In addition, multiple reports have indicated that PTCA is replacing CABG among broad populations of patients requiring coronary revascularization, and CABG is being performed more frequently among higher risk patients [10]. Based on the panel's responses, it is likely that a trend away from CABG towards PTCA will continue in Canada, and will be augmented by the availability of DES as a treatment option.

There are a number of limitations associated with this study. The panel members were recruited from academic medical centers and thus may be more familiar with and more likely to use newer technologies. This may limit the generalizability of the rates provided by the panel to the overall population of Canadian cardiologists and may explain the differences between the panel's estimates and those of the CCN. The study panel was also relatively small; this small sample size may result in estimates that are subject to change if a larger population of cardiologists is surveyed.

Despite these limitations, the results of the panel questionnaire indicate that DES will be an important treatment option for Canadian CAD patients, both among patients currently receiving bare metal stents and for patients currently undergoing CABG surgery. It is difficult to assess the validity of these results, as they relate to future events. A recent report by Poses et al. indicated that physicians were likely to underestimate survival for medically managed CAD patients and overestimate the benefits for such procedures [11]. If this finding applies to the present study, then the rate of DES adoption may be lower than that reported. However, other reports have suggested that coronary revascularization procedures are currently underused, with resulting adverse clinical outcomes [12]. Even if the adoption rates are lower than the projected values presented in Tables 3 through 6, DES is likely to be a commonly used treatment modality. Further, the availability of this less invasive yet more efficacious treatment option may address the potential underuse issues, resulting in greater adoption rates than reported by the panel. The recommended adoption rates presented in Table 7 may then be more realistic estimates for the future use of DES.

Little information is available regarding the impacts of "converting" patients from CABG to stent implantation. Lee et al. evaluated in the impact of bare metal stent use among patients who were at high operative risk or refused CABG [13]. In the Lee et al. study, stent implantation was reported to be safe and clinically beneficial [13]. Use of DES as a treatment option is likely to improve clinical outcomes while maintaining the safety of this less invasive revascularization approach.

We requested information separately for the projected use of DES among individuals with diabetes. Previous studies have reported that CAD patients with diabetes have better outcomes following CABG than with PTCA [14,15]. Available data also suggest that use of bare metal stents improves outcomes among patients with diabetes compared to angioplasty alone [16], although it is unclear whether or not diabetics have worse outcomes following stenting than do non-diabetics [7,17]. While few published data are yet available regarding outcomes among individuals with diabetes following DES implantation, reductions in subsequent restenoses and revascularizations in the general population receiving DES may also occur in the diabetic population. The cardiologist panel felt that DES would become a frequently used treatment option in this population, with adoption rates surpassing those of the overall CAD population.

Comparing the estimated proportion of patients who the panel indicated were likely to receive DES versus the proportion the panel reported "should" receive DES provides interesting findings. The proportion of bare metal stent patients that the panel indicated should receive DES (Table 7) is greater than the proportion who are likely to receive DES (Table 3) for each of the first five years following approval. These estimated proportions become approximately equal at five years following approval (85.0% likely to receive DES, 86.8% should receive DES). A number of factors may influence the difference in proportions between patients who are "likely to" versus "should" receive DES, such as available funding and attitudes towards adoption of new technologies. The estimated proportion of CABG patients who should receive DES (Table 7) is greater than the proportion that are likely to receive DES (Table 5) during the first two years following approval. For years three through five, the "likely to" and "should" proportions are approximately equal for the CABG population. This more rapid convergence of projected rates may reflect the perceived benefits of the less invasive stenting with DES compared to CABG as well as the potential cost savings from DES versus CABG.

A number of reports have indicated that the rate of coronary revascularization procedures in Canada is less than that in the U.S. Bourassa et al. reported that more anginal symptoms were present in Canadian patients prior to revascularization compared to U.S. patients, although Canadian patients apparently experienced greater improvements in quality of life following revascularization procedures [18]. The availability of DES as a treatment option in Canada may change the threshold at which revascularization procedures are considered. The projected uptake rates presented in Tables 3 through 6 certainly indicate that DES is likely to be used for a substantial proportion of revascularization procedures. It will therefore be important to evaluate the impact of this new technology on patient-reported outcomes, such as satisfaction with treatment, satisfaction with the medical care system (e.g., time until treatment), and change in health-related quality of life. These metrics will help to assess further the potential benefits of DES in Canada.

## Conclusions

Cardiologists at tertiary care hospitals in Canada expect the use of drug-eluting stents (DES) for coronary artery revascularization to increase over the next five years. DES will both be used instead of bare metal stents and an alternative to CABG surgery. This increase in DES use will increase initial procedure-related costs compared to bare metal stents, but is likely to decrease subsequent costs due to the decreased need for repeat revascularizations. Medical care decision makers and planners need to prepare for this increased use, in terms of both facility and budget allocation as well as staffing availability and training.

## Competing interests

This study was performed under a research contract from Boston Scientific Corporation. MH has received research funding from Boston Scientific. ML, MAC, and MV are employees of Boston Scientific Corporation. Boston Scientific is providing the article-process charge for this manuscript. Boston Scientific holds a number of patents on the TAXUS Express2 Paclitaxel-Eluting Coronary Stent System. Publication of this article may result in increased consulting work for Exponent, Inc.

## Authors' contributions

MH directed the overall study and participated in all aspects. ML and MV participated in study design and questionnaire development. MAC participated in data analysis. All authors participated in preparation of the manuscript and read and approved the final manuscript.

## Pre-publication history

The pre-publication history for this paper can be accessed here:


